# The Foundations of Corporate Strategies: Comment on "‘Part of the Solution’: Food Corporation Strategies for Regulatory Capture and Legitimacy"

**DOI:** 10.34172/ijhpm.2022.7174

**Published:** 2022-04-05

**Authors:** William H. Wiist

**Affiliations:** Global Health Program, College of Public Health and Human Sciences, Oregon State University, Corvallis, OR, USA.

**Keywords:** Corporate Political Activities, Non-market Strategies, Democracy, Corporate Rights, United States, Commercial Determinants of Health

## Abstract

The "Part of the Solution" article describes how the food industry has evolved its strategies to respond to critics and government regulation by co-option and appeasement to create a less hostile environment. Rather than focusing research on single industries it would be more efficient and productive to focus on corporate political activities (CPAs) that directly influence democratic institutions and processes having authority over laws, policy, rules and regulations that govern industry. The most influential and direct CPA are election campaign donations, lobbying, and the reverse revolving door (RRD). In the United States those CPA flow from rights of corporations that underlie all industry strategies. The US history of how corporations obtained their rights is described, and research about the affirmative effects of those three CPA is summarized. Health research is needed about those CPA and their effects on health law, policy and regulation in the United States and other nations.

Lacy-Nichols and Williams^1^ provided an extensive list of market and non-market strategies the food industry uses to counter health critics and government regulatory efforts. They usefully focus their analysis on the industry’s agile and responsive shift to “part of the solution” strategies based on regulatory responses and capture, relationship building and new market strategies. They describe how industry seeks to co-opt, appease and create an environment less hostile to business interests. The authors show how the industry promotes self-regulation, cultivates partnerships with credible stakeholders, and changes product portfolios to more closely align with health recommendations. Importantly, their description emphasizes that industries evolve in their strategies, and colonize processes, discourses and institutions.

 Their review is similar to other enumerations of the multiple strategies used by single industries. Such reports, along with similar country-by-country identification of strategies, imply the need for separate interventions on multiple strategies for each industry and country. An alternative, more efficient and productive use of limited research and intervention resources would focus on proximal corporate political activities (CPAs) that directly influence democratic government institutions and processes that hold legal authority to establish, implement and enforce laws, policies, regulations and rules that govern industry. The following description of the origins and enumeration of corporate rights and the resultant CPAs in the United States explicates that approach, and is applicable to other democratically governed nations.

## US Geneses of Corporate Strategies

A pro-business bias was present in the United States from the initial European colonization of North America. The colonies themselves were corporations with investors, and their indentured servants, the colonists.^2,3^ (and soon, enslaved Africans). The abrogation of rights that were in colonial corporate charters stimulated the American Revolution, and those rights were reflected in the US Constitution and Bill of Rights.^2,3^ Colonial industries of black enslavement (eg, tobacco) and the commerce in stolen Native American’s land was fundamental to the nation’s development and integral to the lives of the nation’s founders. The underlying business ideologies of profit and race evolved through the War Against the Confederacy into the Gilded Age of extreme corporate power and wealth inequalities. They propagated in the post-World War I and II eras through prominent corporate leaders’ resistance to labor unions, racial integration, and social welfare programs, and their anti-communism. In the mid-twentieth century corporations began using propaganda to instill in the public consciousness the identification of free-enterprise with democracy and equating government interventions with tyranny and oppression.^4^ Later, business solidified its hold on society with strategic initiatives,^5^ the financial sector’s policy role,^6^ and the influence of the CEO’s role.^7^

Across US history the Congress, state legislatures, and especially the Supreme Court, established corporate rights (Figure). The Court based its decisions about corporate rights on: (*a*) the rights (eg, first Amendment freedom of speech) of the people behind the corporate entity (“piercing the veil”) and (*b*) rights of corporate personhood. However, corporate rights were achieved less from Court decisions about the rights of the corporate entity than from larger fights for the rights for humans (eg, free speech), especially those of enslaved and free Blacks (ie, the 14th Amendment), and from long-running disputes over federal versus state control of business rights and rights of African-Americans.^2,8^

**Figure:**
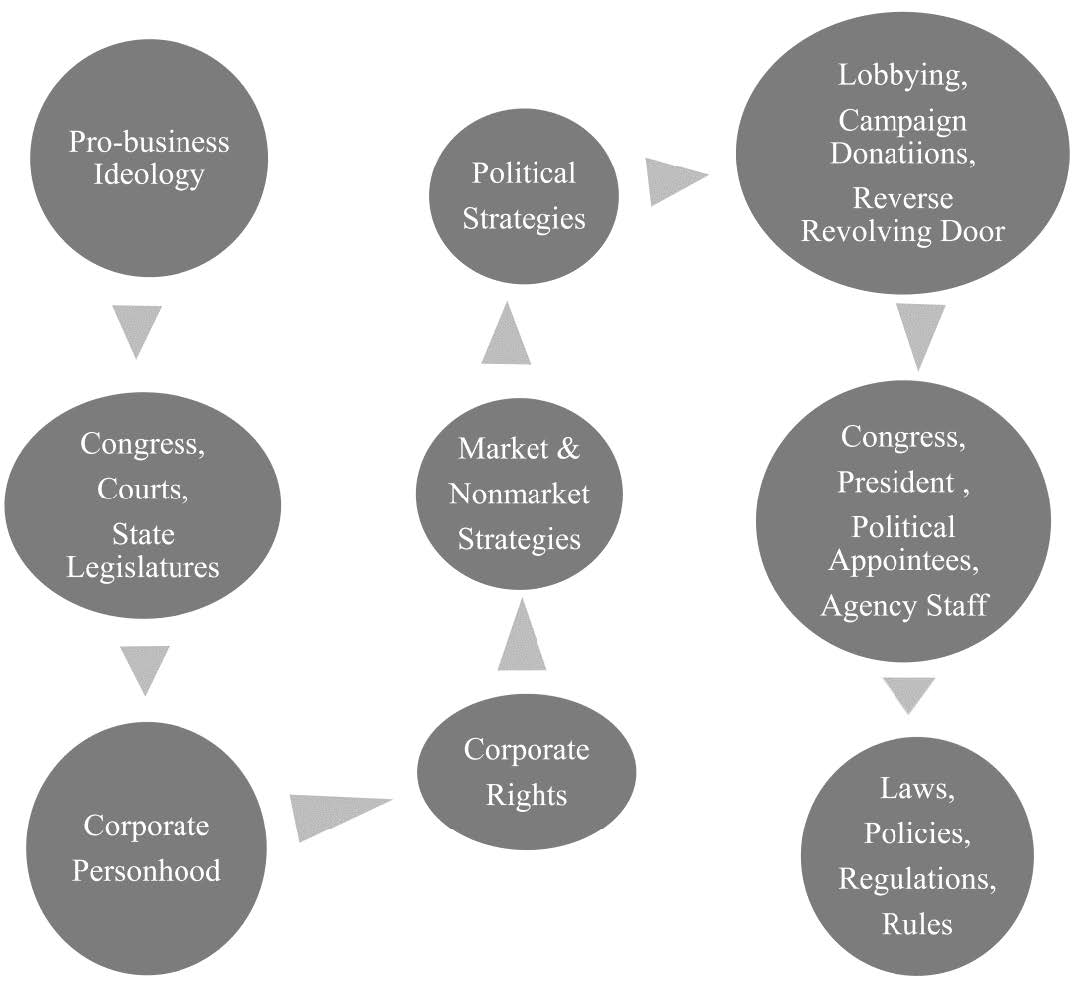
Pathway of Corporate Influence on Governing Institutions and Processes.

 Rights awarded to corporations include the right to sue and be sued; diversify and be integrated with other corporate units; own stock; initiate and sign contracts; have equal protection under the law and due process; freedom from unreasonable searches and seizure; compensation for government takings; jury trial in criminal and civil cases; freedom from double jeopardy; freedom from excessive fines; commercial speech; political speech; freedom of religion; to sue governments for loss of anticipated profits; shareholders subordinate to management; shareholder limited liability; and an unlimited lifespan. These rights underlie and enable all industry marketing and non-marketing strategies.

## Democracy

The essence of democracy is that all citizens should have an equal voice so that political institutions are as responsive as possible to the interests and values of citizens,^9^ and it is in elections that democracy comes the closest to equality.^10^ Most people assume that the pursuit of equity is a major duty of government^11^ but corporation’s wealth gives them disproportionate access, preference, and influence^12^ and distorts policy-makers’ work toward those who can afford the cost.^13^ That distortion makes government less responsive to the average constituent, whose policy positions are negatively related to those of business.^14^ That undermines citizens’ trust in their government^12^ and leads them to view politicians as corrupt^15^ and, correctly, that ordinary citizens have little influence on policy.^14^

## Direct Political Strategies

A wide range of practices have been categorized as CPA; some shape the opinion environment, others directly influence policy makers.^12^ Three CPA are the most powerful and important because they are most proximal to the corporate rights that underlie industry strategies, and they are aimed directly at influencing democratic government institutions’ and processes’ (Figure) that have legal authority over corporations: (1) lobbying of legislative and executive branches of government, (2) donations to election campaigns, and (3) the “reverse revolving door” (RRD) (former corporate officials politically appointed to government positions with policy, decision-making and regulatory authority over industry). Each of the three CPA is complex, with many points of opportunity for industry to advance their agenda or to thwart policy change.^11^

The empirical research findings about the effects of CPA activities on policy outcomes are mixed.^16^ Recently an increased study of political activities suggests their influences on health issues such as government efforts to control obesity and noncommunicable diseases in Thailand,^17^ self-regulation of food marketing to children in Malaysia,^18^ food marketing policies in Brazil,^19^ commercial milk formula policy in the Philippines^20^ and food marketing in South Africa, Columbia and Indonesia.^21^ However, there has been little empirical health research into the three direct CPA discussed here.

 Some countries, including the United States, have lobbyist registration and reporting requirements, laws regarding reporting of election campaign donations, and ethics codes and reporting rules for lobbyists, politicians, government officials and employees. Although the policies and procedures need greater transparency, public reporting, enforcement and data access, they have provided some data for research such as that cited below. Space limitations allow for only a brief description of key aspects of each of the three CPA and brief summaries of research findings.

## Lobbying

Industry spends millions of dollars annually on lobbying^22^ conducted by employees, outside contractors or by industry associations. Many lobbyists are former congressional members or staff. Lobbying targets Congress and the executive branch, political appointees, staff who review legislation and regulations, and bureaucrats who write and enforce rules. Lobbyists subsidize strategically selected government officials with limited time and resources^13^ by serving as expert advisors to draft legislative bills, propose amendments, draft speeches, provide research reports or testimony, serve on advisory committees, assist in writing rules, build coalitions, mobilize constituencies, host fundraisers and donate to election campaigns and to politicians’ affiliated foundations.

Most lobbying is conducted in low visibility settings, without lobbyists’ activity being recorded – before legislation reaches the voting stage, during the formulation of new policies, moving policy through committees (where procedures may be as important as position preferences), working on amendments, garnering support from other members of Congress, and in the day-to-day informal cultivation of friendly relationships with committee chairs and key members. Much lobbying is directed at regulatory agencies, particularly when an agency has requested public comments on proposed rules. Lobbyists are often the only ones to appear to testifying before regulatory committees.^11^

Industry lobbying bends policy toward industry preferences^21^ and away from the preferences of the average citizen.^11^ It can substantially benefit corporate financial returns, reduce effective corporate tax rates, shape deregulation policies, influence restrictions against unionization, influence marketing regulations, and influence healthcare expenditures, policies, and laws.^23,24^

## Election Campaign Donations

The US political system is money-driven so candidates spend a large proportion of their time fund-raising. Elections are the one direct threat to industry power over government^10^ so corporations and their wealthy officers and employees, and corporate earnings-derived charitable foundations^25^ contribute billions of dollars^10^*indirectly* to individual candidate’s election campaigns and political parties and their conventions, and *directly* to campaigns through political action committees (PACs), Super PACs, and certain types of nonprofit organizations that do not have to identify donors (“dark money”). They also donate to ballot initiative campaigns. Members of congressional committees with industry-relevant policy expertise and jurisdiction over an industry are especially targeted by donors from those industries. Some countries (eg, Belgium, Canada, France, the United States) have bans on corporate donations directly to political parties and candidates.

Election campaign contributions give donors access to politicians to influence priorities and offer help, thereby creating obligatory bonds.^25^ The more money a politician receives the more they are likely to give time and effort on the donor’s behalf by speaking for their interests, adding amendments to a bill and showing up at committees to vote.^10^ Thus, corporations have disproportionate influence compared to the majority of average citizens who cannot afford to contribute significant amounts.^26^

Donations to election campaigns increase the award of government contracts^27^ and corporate profits, influence pro-business government spending and legislators’ positions,^28^ and votes favorable to corporations.^29^ Corporations that make large political donations tend to be less compliant with regulations.^23^ Politicians who receive the most corporate PAC money are more likely to vote favorably toward the contributing industry.^30^ Contributions can affect safety inspections and citations for violations,^27^ promote congressional advocacy of industry and support for pro-business spending programs.^28^ Corporate donations can also signal bureaucrats that regulatory enforcement may be troublesome.^27^ Campaign funding especially goes to members of committees with industry-relevant policy expertise and jurisdiction of the contributing industry. Donations are likely to carry influence earlier and in less scrutinized, subtle legislative steps than highly visible votes.^31^

## The Reverse Revolving Door

A US president makes thousands of political appointments of individuals from the private sector, many from corporations, to policymaking or decision-making positions in government as an agency head or other senior administrative positions, to regulatory commissions, advisory committees, boards or councils that have influence or authority over industry (RRD). Also, former corporate employees or lobbyists are frequently hired to staff congressional committees or member’s offices. The corporate conflicts of interest of “reverse revolvers” may automaticallyand unconsciously^32^ influence their independence and their objectivity, leading them to biases in the formulation, adoption, and implementation of laws, policies and regulations to favor industry. Those biases may stem from: (*a*) reciprocity, the societally normalized internal belief that there is an obligation to reciprocate a favor with a favor,^33^ and (*b*) a corporate orientation developed over a lifetime which can prevent them from completely separating their industry and government roles.^34^

Some of the RRD benefits corporations receive include government bail-outs,^35^ increased procurement contracts,^36^ more lenient patent reviews,^37^ more deregulatory reforms,^38^ and increased revenue and profits^39^; certification of safety compliance without required testing,^40^ exclusion of health and sanitation provisions from standards,^41^ and more lax regulation.^42^

## Priorities for Future Research

Research into the influence of corporations on health would lead to greater progress for policy and advocacy if the research and professional education curricula emphasized industry’s political activities aimed directly at democratic institutions and processes having legal authority over corporations. That work can build on social science theories, methods and data sources about CPA^43^ to seek answers to health research questions about the effects of those activities on health law, policy, regulation, infrastructure, funding, programs and services, and population health^44^ and natural environment outcomes.

Although the focus here is three CPA in the United States they also occur in other high income democratically governed nations (eg, European Union, United Kingdom, Canada, Australia, and Japan).^45-49^ Health researchers in undemocratically governed countries or emerging economies need to investigate the genesis of corporate rights and resultant predominant types of CPA.^47^ In countries without strong institutions and systems of checks and balances, including public reporting on CPA, research methods used in other countries^19^ such as interviews with relevant individuals, news reports, websites, and other data sources can be used to study CPA.

Results of public health research on CPA will have implications for policy and advocacy. Policies and procedures must be strengthened for managing conflict of interests for researchers,^50^ health organizations^51^ and public officials,^52^ election campaign finance^53^ and lobbying registration and disclosure.^48^ Reclaiming democracy from CPA power will require a broad coalition of advocates with diverse interests unified on establishing a new balance of power to ensure that corporations exist for the public good, the people, and democracy^8^; to recreate corporations as agents of opportunity rather than recipients of privilege, to curb their potentially dangerous power and limit their contribution to inequalities^3^ in wealth and racial justice.

 Alternatively to the three CPA research priorities proposed above, researchers who continue to prioritize studying single industry strategies should expand research to industries contributing to the most serious current threats to health and democracy, such as climate change (ie, fossil fuel industries), income and wealth inequality (ie, financial industries), war (ie, “defense” industries), autocratic and violent ideologies, false information, prejudice, and invasion of privacy, (eg, digital media industries), and the normalization of the pro-business ideology (ie, TV, film and publishing industries, and higher education).

 The “Part of the Solution” article acknowledged election campaign funding, lobbying and the revolving door among the many industry non-market strategies used to manipulate policy-making and policy-makers. It identified the food industry’s adaptation to environmental conditions through appeasement, co-option and partnership as a form of consensual and socially legitimate power. This commentary focuses on how in the US industry came to have that power and exercise it, and in contrast to “Part of the Solutions,” proposes research priorities from among industry strategies. Health researchers are urged to move forward from the repetitive examination of single industry strategies and study the underlying foundations from which industry gains its power to employ whatever exiting or adaptive “solution” strategies it chooses. Research and advocacy could be more substantively and efficiently advanced by prioritizing the three CPA recommended herein rather than continuing the enumeration of industry strategies as in “Part of the Solution.” There are challenges to achieving that priority but it is imperative to overcome them in order to promote health and strengthen democracy.

## Ethical issues

 Not applicable.

## Competing interests

 Textbook royalties; personal financial donations to not-for-profit corporate reform advocacy organizations.

## Author’s contribution

 WHW is the single author of the paper.
